# Supplementary material for: Pancreatic cancer symptom trajectories from Danish registry data and free text in electronic health records
Source: eLife. 2023 Nov 21;12:e84919. doi: 10.7554/eLife.84919 (PMC10662947; doi:10.7554/eLife.84919)
Supplement: Supplementary file 1. [file elife-84919-supp1.docx]

**Supplementary file 1**

**Supplementary file 1a**

Most commonly known pancreatic cancer symptoms

| **Symptom** | **ICD-10 version** | **Reference** |
| --- | --- | --- |
| Jaundice | Unspecified jaundice | [pancreaticcanceraction.org/](http://pancreaticcanceraction.org/), [cancer.org/](http://cancer.org/), [pancan.org/](http://pancan.org/) |
| Abdominal and pelvic pain | Abdominal and pelvic pain | [pancreaticcanceraction.org/](http://pancreaticcanceraction.org/), [cancer.org/](http://cancer.org/), [pancan.org/](http://pancan.org/) |
| Weight loss | Symptoms and signs concerning food and fluid intake | [pancreaticcanceraction.org/](http://pancreaticcanceraction.org/), [cancer.org/](http://cancer.org/), [pancan.org/](http://pancan.org/) |
| Nausea and vomiting | Nausea and vomiting | [pancreaticcanceraction.org/](http://pancreaticcanceraction.org/), [cancer.org/](http://cancer.org/) |
| Enlargened gallbladder | Other diseases of gallbladder | [cancer.org/](http://cancer.org/) |
| Enlargened liver | Hepatomegaly and splenomegaly, not elsewhere classified | [cancer.org/](http://cancer.org/) |
| New-onset diabetes* | Type 2 diabetes mellitus | [pancreaticcanceraction.org/](http://pancreaticcanceraction.org/), [cancer.org/](http://cancer.org/), [pancan.org/](http://pancan.org/) |
| Blood clots | Arterial embolism and thrombosis | [cancer.org/](http://cancer.org/) |
| Fatigue | Malaise and fatigue | [pancreaticcanceraction.org/](http://pancreaticcanceraction.org/) |
| Loss of appetite | Symptoms and signs concerning food and fluid intake/Anorexia | [pancreaticcanceraction.org/](http://pancreaticcanceraction.org/), [pancan.org/](http://pancan.org/) |
| Changes in stool or bowel habit | Change in bowel habit | [pancreaticcanceraction.org/](http://pancreaticcanceraction.org/), [pancan.org/](http://pancan.org/) |
| Indigestion | Functional dyspepsia | [pancreaticcanceraction.org/](http://pancreaticcanceraction.org/) |

* This has not been used in Fig. 1 Venn diagram, since it is in the disease chapter and not the symptoms chapter, which is the one we compare with.

**Supplementary file 1b**

Complete names of ICD-10 codes that have been shortened for overview.

| **Abbreviation** | **Complete name** |
| --- | --- |
| Abnormal findings on diagnostic images | Abnormal findings on diagnostic images of other body structures |
| Primary hypertension | Essential primary hypertension |
| Irregular menstruation | Excessive, frequent and irregular menstruation |
| Hearing loss | Other hearing loss |
| Digestive system and abdomen | Systems and signs involving digestive system and abdomen |
| Fever | Fever of other and unknown origin |
| Hyperalimentation | Other hyperalimentation |
| Pain | Pain, not elsewhere classified |
| Intestinal disorders | Other functional intestinal disorders |
| Oedema | Oedema, not elsewhere classified |
| Anaemia | Other anaemias |
| Jaundice | Unspecified jaundice |
| Paralytic ileus and intestinal obstruction | Paralytic ileus and intestinal obstruction without hernia |
| Haemorrhage | Haemorrhage, not elsewhere classified |
| Intestinal disorders | Other functional intestinal disorders |
| Abnormal blood pressure | Abnormal blood-pressure reading, without diagnosis |
| Soft tissue disorders | Other soft tissue disorders, not elsewhere classified |
| Elevation of levels of transaminase and LDH | Elevation of levels of transaminase and lactic acid dehydrogenase (LDH) |
| Food and fluid intake | Symptoms and signs concerning food and fluid intake |
| Emotional states symptoms | Symptoms and signs involving emotional states |
| Unspecified haematuria | Haematuria |
| Urinary system | Other symptoms and signs involving the urinary system |
| Unspecified abdominal hernia | Abdominal hernia |
| Porphyrin and bilirubin metabolism | Disorder of porphyrin and bilirubin metabolism |
| Functional capacity | Findings related to functional capacity |
| Findings on diagnostic imaging of lung | Abnormal findings on diagnostic imaging of lung |
| Findings on diagnostic imaging of other body structure | Abnormal findings on diagnostic imaging of other body structure |
| Localized swelling, mass and lump of skin | Localized swelling, mass and lump of skin and subcutanous tissue |
| Abnormal function studies | Abnormal results of function studies |

**Supplementary file 1c**

The most frequent symptoms, p-values, odds ratio and 95% confidence interval found by text mining clinical notes and analysed by a logistic regression model.

| **Symptom** | **Odds ratio** | **p-value** | **Lower CI** | **Upper CI** | **Number of patients** |
| --- | --- | --- | --- | --- | --- |
| Pain, not elsewhere classified | 1.24 | 9.18E-06 | 1.13 | 1.36 | 2126 |
| Nausea and vomiting | 2.16 | 1.08E-79 | 1.00 | 2.34 | 1366 |
| Abdominal and pelvic pain | 3.76 | 2.59E-204 | 3.46 | 4.00 | 1063 |
| Symptoms and signs concerning food and fluid intake | 4.74 | 2.34E-263 | 4.34 | 5.18 | 1014 |
| Unspecified jaundice | 19.8 | 0 | 17.7 | 22.1 | 962 |
| Fever of other and unknown origin | 1.59 | 3.75E-21 | 1.45 | 1.75 | 614 |
| Paralytic ileus and intestinal obstruction without hernia | 33.0 | 2.97E-249 | 27.7 | 41.7 | 408 |
| Other functional intestinal disorders | 1.52 | 1.20E-09 | 1.33 | 1.74 | 278 |
| Ascites | 4.39 | 9.77E-84 | 3.7877 | 5.0004 | 271 |
| Flatulence and related conditions | 2.24 | 1.29E-23 | 1.91 | 2.62 | 206 |
| Disorders of porphyrin and bilirubin metabolism | 20.0 | 4.26E-123 | 15.6 | 25.7 | 196 |
| Hepatomegaly and splenomegaly, not elsewhere classified | 3.32 | 9.44E-44 | 2.80 | 3.93 | 193 |
| Hyperhidrosis | 2.82 | 2.83E-32 | 2.38 | 3.35 | 180 |
| Abnormal results of function studies | 1.69 | 8.18E-10 | 1.43 | 1.99 | 176 |
| Heartburn | 1.44 | 1.68E-05 | 1.22 | 1.70 | 171 |
| Other diseases of pancreas | 10.6 | 3.17E-83 | 8.33 | 13.4 | 148 |
| Acute pancreatitis | 8.05 | 4.98E-15 | 4.77 | 13.6 | 27 |

**Supplementary file 1d**

Registry-based symptoms trajectories listing the number of patients following each trajectory and the median survival in days for each group. Only trajectories with at least 100 patients in a trajectory are shown here.

| **Symptom 1** | **Symptom 2** | **Symptom 3** | **Patient counts** | **Survival in days**  **(median)** | **Percent of total PC cases (%)** |
| --- | --- | --- | --- | --- | --- |
| Malignant neoplasm of pancreas | Ascites | Nausea and vomiting | 109 | 229 | 1.24 |
| Malignant neoplasm of pancreas | Fever of other and unknown origin | Nausea and vomiting | 212 | 443 | 2.39 |
| Syncope and collapse | Malignant neoplasm of pancreas | Nausea and vomiting | 114 | 165 | 0.89 |
| Unspecified haematuria | Malignant neoplasm of pancreas | Nausea and vomiting | 113 | 230 | 1.24 |
| Pain in throat and chest | Malignant neoplasm of pancreas | Nausea and vomiting | 105 | 183 | 0.99 |
| Abdominal and pelvic pain | Malignant neoplasm of pancreas | Nausea and vomiting | 585 | 195 | 1.05 |
| Abdominal and pelvic pain | Malignant neoplasm of pancreas | Ascites | 210 | 227.5 | 1.23 |
| Malignant neoplasm of pancreas | Fever of other and unknown origin | Ascites | 102 | 442 | 2.39 |
| Abdominal and pelvic pain | Malignant neoplasm of pancreas | Fever of other and unknown origin | 241 | 334 | 1.80 |
| Pain in throat and chest | Malignant neoplasm of pancreas | Pain, not elsewhere classified | 170 | 217 | 1.17 |
| Other symptoms and signs involving the urinary system | Malignant neoplasm of pancreas | Pain, not elsewhere classified | 126 | 189.5 | 1.02 |
| Haemorrhage from respiratory passages | Malignant neoplasm of pancreas | Pain, not elsewhere classified | 123 | 179 | 0.97 |
| Unspecified haematuria | Malignant neoplasm of pancreas | Pain, not elsewhere classified | 189 | 179 | 0.97 |
| Other symptoms and signs involving the digestive system and abdomen | Malignant neoplasm of pancreas | Pain, not elsewhere classified | 116 | 210.5 | 1.14 |
| Dizziness and giddiness | Malignant neoplasm of pancreas | Pain, not elsewhere classified | 111 | 174 | 0.94 |
| Malignant neoplasm of pancreas | Fever of other and unknown origin | Pain, not elsewhere classified | 377 | 419 | 2.26 |
| Abdominal and pelvic pain | Malignant neoplasm of pancreas | Pain, not elsewhere classified | 1057 | 189 | 1.02 |
| Syncope and collapse | Malignant neoplasm of pancreas | Pain, not elsewhere classified | 207 | 151.5 | 0.82 |
| Unspecified jaundice | Fever of other and unknown origin | Pain, not elsewhere classified | 106 | 544 | 2.94 |
| Malignant neoplasm of pancreas | Ascites | Pain, not elsewhere classified | 191 | 236 | 1.27 |
| Abdominal and pelvic pain | Malignant neoplasm of pancreas | Malaise and fatigue | 676 | 197 | 1.06 |
| Pain in throat and chest | Malignant neoplasm of pancreas | Malaise and fatigue | 115 | 233.5 | 1.26 |
| Malignant neoplasm of pancreas | Fever of other and unknown origin | Malaise and fatigue | 249 | 419 | 2.26 |
| Syncope and collapse | Malignant neoplasm of pancreas | Malaise and fatigue | 116 | 170 | 0.92 |
| Unspecified haematuria | Malignant neoplasm of pancreas | Malaise and fatigue | 128 | 187 | 1.01 |
| Malignant neoplasm of pancreas | Ascites | Malaise and fatigue | 131 | 254 | 1.37 |
| Abdominal and pelvic pain | Malignant neoplasm of pancreas | Symptoms and signs concerning food and fluid intake | 434 | 208 | 1.12 |
| Malignant neoplasm of pancreas | Fever of other and unknown origin | Symptoms and signs concerning food and fluid intake | 168 | 471 | 2.54 |
| Malignant neoplasm of pancreas | Fever of other and unknown origin | Cachexia | 111 | 419 | 2.26 |
| Abdominal and pelvic pain | Malignant neoplasm of pancreas | Cachexia | 268 | 216 | 1.17 |
| Syncope and collapse | Malignant neoplasm of pancreas | General disability | 135 | 148 | 0.80 |
| Unspecified haematuria | Malignant neoplasm of pancreas | General disability | 145 | 202 | 1.09 |
| Malignant neoplasm of pancreas | Fever of other and unknown origin | General disability | 281 | 427.5 | 2.31 |
| Pain in throat and chest | Malignant neoplasm of pancreas | General disability | 134 | 204 | 1.10 |
| Malignant neoplasm of pancreas | Ascites | General disability | 129 | 256 | 1.38 |
| Other symptoms and signs involving the urinary system | Malignant neoplasm of pancreas | General disability | 110 | 234 | 1.26 |
| Abdominal and pelvic pain | Malignant neoplasm of pancreas | General disability | 744 | 201 | 1.09 |
| Abdominal and pelvic pain | Malignant neoplasm of pancreas | Other ill-defined and unspecified causes of mortality | 131 | 74 | 0.40 |
| Malignant neoplasm of pancreas | Pain, not elsewhere classified | Other ill-defined and unspecified causes of mortality | 142 | 185 | 1.00 |

**Supplementary file 1e**

Text-mined symptom trajectories listing the number of patients following each trajectory and the median survival in days for each group. The table lists all symptom trajectories followed by a minimum of 20 patients.

| **Symptom 1** | **Symptom 2** | **Symptom 3** | **Survival in days (median)** | **Patient counts** | **Percent of total PC cases (%)** |
| --- | --- | --- | --- | --- | --- |
| Abnormal blood-pressure reading, without diagnosis | Abdominal and pelvic pain | Disorders of porphyrin and bilirubin metabolism | 225 | 28 | 0.91 |
| Pain, not elsewhere classified | Abdominal and pelvic pain | Disorders of porphyrin and bilirubin metabolism | 140 | 41 | 1.33 |
| Abnormalities of heart beat | Unspecified jaundice | Disorders of porphyrin and bilirubin metabolism | 119.5 | 20 | 0.65 |
| Abnormal blood-pressure reading, without diagnosis | Unspecified jaundice | Disorders of porphyrin and bilirubin metabolism | 207.5 | 53 | 1.72 |
| Cough | Unspecified jaundice | Disorders of porphyrin and bilirubin metabolism | 124 | 32 | 1.04 |
| Pain in throat and chest | Unspecified jaundice | Disorders of porphyrin and bilirubin metabolism | 177 | 20 | 0.65 |
| Dizziness and giddiness | Unspecified jaundice | Disorders of porphyrin and bilirubin metabolism | 124 | 38 | 1.23 |
| Pain, not elsewhere classified | Unspecified jaundice | Disorders of porphyrin and bilirubin metabolism | 128 | 75 | 2.44 |
| Abnormal blood-pressure reading, without diagnosis | Symptoms and signs concerning food and fluid intake | Disorders of porphyrin and bilirubin metabolism | 120.5 | 31 | 1.01 |
| Pain, not elsewhere classified | Symptoms and signs concerning food and fluid intake | Disorders of porphyrin and bilirubin metabolism | 134 | 46 | 1.49 |
| Abnormal blood-pressure reading, without diagnosis | Abdominal and pelvic pain | Paralytic ileus and intestinal obstruction without hernia | 107 | 35 | 1.14 |
| Pain, not elsewhere classified | Abdominal and pelvic pain | Paralytic ileus and intestinal obstruction without hernia | 91 | 61 | 1.98 |
| Abnormalities of heart beat | Unspecified jaundice | Paralytic ileus and intestinal obstruction without hernia | 132 | 22 | 0.71 |
| Abnormal blood-pressure reading, without diagnosis | Unspecified jaundice | Paralytic ileus and intestinal obstruction without hernia | 190.5 | 58 | 1.88 |
| Cough | Unspecified jaundice | Paralytic ileus and intestinal obstruction without hernia | 53 | 43 | 1.40 |
| Pain in throat and chest | Unspecified jaundice | Paralytic ileus and intestinal obstruction without hernia | 123 | 22 | 0.71 |
| Polyuria | Unspecified jaundice | Paralytic ileus and intestinal obstruction without hernia | 255 | 22 | 0.71 |
| Dizziness and giddiness | Unspecified jaundice | Paralytic ileus and intestinal obstruction without hernia | 124 | 47 | 1.53 |
| Pain, not elsewhere classified | Unspecified jaundice | Paralytic ileus and intestinal obstruction without hernia | 107 | 103 | 3.35 |
| Abnormal blood-pressure reading, without diagnosis | Unspecified jaundice | Fever of other and unknown origin | 243 | 43 | 1.40 |
| Cough | Unspecified jaundice | Fever of other and unknown origin | 93.5 | 25 | 0.81 |
| Pain in throat and chest | Unspecified jaundice | Fever of other and unknown origin | 115 | 20 | 0.65 |
| Dizziness and giddiness | Unspecified jaundice | Fever of other and unknown origin | 147.5 | 32 | 1.04 |
| Pain, not elsewhere classified | Unspecified jaundice | Fever of other and unknown origin | 104 | 74 | 2.40 |
| Abnormal blood-pressure reading, without diagnosis | Unspecified jaundice | Abnormal results of function studies | 128 | 22 | 0.71 |
| Dizziness and giddiness | Unspecified jaundice | Abnormal results of function studies | 94 | 20 | 0.65 |
| Pain, not elsewhere classified | Unspecified jaundice | Abnormal results of function studies | 74.5 | 38 | 1.23 |
